# Impact of white tea extract - enriched calcium silicate cements on the flexural Strength and collagen degradation of dentin

**DOI:** 10.6026/9732063002001132

**Published:** 2024-09-30

**Authors:** Sufia Parveen, Pragy SJ, Alakesh Singha, Deeksha Goel, Subasish Behera, Soumyaranjan Nanda

**Affiliations:** 1 Department of Conservative Dentistry and Endodontics, Buddha Institute of Dental Sciences and Hospital, Patna, Bihar, India; 2 Department of Dentistry, ABVM Government Medical College, Rajnandgaon, Chhattisgarh, India; 3 Private Practitioner, Department of Conservative Dentistry and Endodontics, Alaka's Odontocare Dental clinic & Endodontic Centre, Agartala, Tripura, India; 4 Department of Conservative Dentistry and Endodontics, DJ College of Dental Science and Hospital, Niwari Road, Modinagar, U.P., India; 5 Department of Conservative Dentistry and Endodontics, Government Dental College and Hospital (SCB), Manglabag, Cuttack, Odisha, India; 6 Private Dental Practitioner, Conservative Dentistry and Endodontics, Cuttack, Odisha, India

**Keywords:** White tea extract, calcium silicate cement, dentin, flexural strength, hydroxy-proline release, restorative dentistry

## Abstract

The flexural strength of dentin and the rate at which dentin collagen degrades are two important variables that affect how long restorative dental materials last. The use of natural extracts to improve the qualities of dental materials has been studied recently. The antioxidant-rich white tea extract has the potential to be beneficial when mixed with calcium silicate cements. This in vitro research used forty removed human molar teeth. After the teeth were sectioned to obtain dentin specimens, the dentin was randomly divided into four groups (n=10) according to the type of treatment: Group A (control) consisted of the dentin that had not been treated; Group B treated the dentin using conventional calcium silicate cement; Group C treated the dentin using calcium silicate cement combined with 5% white tea extract; and Group D treated the dentin using calcium silicate cement combined with 10% white tea extract. A universal testing apparatus was used to measure flexural strength, and hydroxy-proline release analysis was used to measure collagen degradation during a 30-day period. ANOVA and post-hoc tests were used to examine the data, with a significance level of p < 0.05.Incorporating white tea extract into calcium silicate cements improves the flexural strength and lowers collagen degradation of dentin. The benefits are more noticeable with larger concentrations of extract.

## Background:

The overall strength and longevity of dental restorations are greatly influenced by the integrity of the dentin, especially its collagen matrix [1]. Over time, dental restorations may fail due to the mechanical qualities of dentin being compromised by collagen degradation, which is often made worse by restorative operations [2]. Because of its advantageous characteristics, including as their bioactivity, biocompatibility, and capacity to stimulate dentin regeneration, calcium silicate cements (CSCs) have found extensive use in restorative dentistry [3]. But one possible tactic to improve CSC function even more is to add natural extracts with antioxidant qualities to them [4]. Strong antioxidant and anti-inflammatory characteristics of white tea extract, which is high in polyphenols and flavonoids, may shield dentin collagen from enzymatic degradation [5]. Antioxidants have been shown in earlier research to suppress matrix metallo-proteinases (MMPs) that break down collagen, protecting dentin's structural integrity [6]. Thus, the purpose of this work is to examine how the incorporation of white tea extract into CSCs affects dentin's flexural strength and collagen degradation.

## Materials and Methods:

## Specimen Preparation:

For this in vitro investigation, forty recently removed human third molars free of cavities and restorations were chosen. Before being used, the teeth were kept in a 0.9% saline solution at 4°C. Using a low-speed diamond saw with water cooling, each tooth was sectioned transversely at the cementoenamel junction to create dentin slabs that were 2 mm thick. After that, the dentin slabs were polished to a uniform surface using 600-grit silicon carbide paper.

## Experimental Groups:

Based on the kind of treatment, the specimens were randomly assigned to four groups (n = 10). Group B had dentin treated with standard calcium silicate cement (ProRoot MTA, Dentsply), whereas Group A (Control) contained dentin that had not been treated. Dentin treated with calcium silicate cement and 5% white tea extract was used in Group C, whereas 10% white tea extract was added to dentin treated with the same cement in Group D.

## Calcium Silicate Cement Preparation:

The manufacturer's instructions were followed in the preparation of the calcium silicate cement. White tea extract was added to the cement powder for Groups C and D at a weight percentage of 5% and 10%, respectively. In order to create the extract powder, white tea leaves were steeped in distilled water at 80°C for ten minutes. The tea leaves were then filtered and lyophilized. To get the cement formulations to the right consistency for applying on the dentin specimens, they were manually combined with distilled water.

## Flexural Strength Testing:

Using a universal testing machine (Instron, USA), a three-point bending test was performed on each dentin slab. The specimens were positioned atop two 10 mm-span supporting rods, and a load was applied at the slab's center at a crosshead speed of 0.5 mm/min until the specimens failed.

## Evaluation of Collagen Degradation:

Over the course of 30 days, the amount of hydroxy-prolin - a sign of collagen breakdown-that was released from the dentin specimens was measured. 5 mL of phosphate-buffered saline (PBS) were used to submerge each specimen, and it was then incubated at 37°C. Every 1, 7, 14, and 30 days, the PBS was collected and changed. Using a colorimetric test (Hydroxy-proline test Kit, Sigma-Aldrich) in accordance with the manufacturer's instructions, the amount of hydroxy-proline in the collected PBS was measured. A micro-plate reader was used to determine absorbance at 560 nm.

## Statistical analysis:

IBM SPSS software, version 25.0, was used to analyze the data. For every group, the flexural strength and hydroxy-proline release mean and standard deviation were determined. To identify significant differences between the groups, a one-way ANOVA with post-hoc Tukey's test was used, with a threshold of statistical significance of p < 0.05.

## Results:

## Flexural strength:

The flexural strength values of the dentin specimens across the different groups are presented in Table 1. The incorporation of white tea extract into calcium silicate cement significantly increased the flexural strength of the dentin compared to the control group and the group treated with conventional calcium silicate cement. Group D (10% white tea extract) exhibited the highest flexural strength, followed by Group C (5% white tea extract), Group B (conventional calcium silicate cement), and Group A (control).

## Collagen degradation:

The hydroxy-proline release values, indicative of collagen degradation, for each group over the 30-day period are presented in Table 2. The groups treated with white tea extract (Groups C and D) showed significantly lower hydroxy-proline release compared to the control group and the group treated with conventional calcium silicate cement. The lowest collagen degradation was observed in Group D (10% white tea extract), followed by Group C (5% white tea extract), Group B (conventional calcium silicate cement), and Group A (control).

## Statistical analysis:

Statistical analysis revealed significant differences in both flexural strength and collagen degradation among the groups (p < 0.05). The flexural strength was significantly higher in Groups C and D compared to Groups A and B, with Group D showing the highest values. Similarly, the hydroxy-proline release was significantly lower in Groups C and D compared to Groups A and B, with Group D exhibiting the lowest values, indicating reduced collagen degradation. These results suggest that the incorporation of white tea extract into calcium silicate cement enhances the mechanical properties of dentin while simultaneously reducing collagen degradation.

## Discussion:

The results of this investigation show that adding white tea extract to calcium silicate cement greatly improves dentin's flexural strength and slows down the deterioration of collagen. These results are in line with other studies that show antioxidant-rich natural extracts may enhance the mechanical qualities of dental materials [1]. The antioxidant activity of the polyphenols in the white tea extract may maintain the collagen matrix and shield it from enzymatic degradation, which explains the observed improvement in flexural strength in the extract-treated groups [2]. Given that the collagen matrix's structural integrity is essential for withstanding mechanical stress, this stabilization probably helps the dentin's mechanical integrity [3]. Furthermore, hydroxy-proline release, a measure of collagen degradation, significantly decreased, indicating that white tea extract efficiently inhibits matrix metallo-proteinases (MMPs), the enzymes that break down collagen in dentin [4]. Antioxidants have been shown in earlier research to be able to inhibit MMP activity, protecting the collagen network in dentin [5]. The effects were more noticeable at the higher concentration of white tea extract (10%), suggesting that the advantages seen were depending on the quantity of extract used. This dose-dependent impact is consistent with other research showing that dental materials with higher antioxidant concentrations might provide more protection [6]. The present findings underscore the potential of white tea extract as a beneficial additive in dental materials, particularly in enhancing the mechanical properties of calcium silicate cement. The significant improvement in flexural strength observed in the extract-treated groups may be attributed to the synergistic interaction between the bioactive compounds in white tea and the calcium silicate matrix. Previous studies have also highlighted the role of natural extracts in enhancing the mechanical properties of biomaterials by interacting with their inorganic components [7]. The poly-phenolic compounds in white tea, known for their strong antioxidant activity, likely facilitate cross-linking within the collagen matrix; thereby reinforcing the structural integrity of dentin [8]. In addition to the mechanical benefits, the reduction in hydroxy-proline release suggests that white tea extract may play a crucial role in preserving the organic components of dentin. The inhibition of matrix metallo-proteinases (MMPs) by the extract could be one of the key mechanisms through which collagen degradation is slowed. This protective effect is consistent with previous studies demonstrating the ability of antioxidants to modulate enzymatic activity, thereby preventing the breakdown of collagenous tissues [9].

The preservation of collagen is particularly important in dental applications, as it contributes to the overall durability and resilience of the dentin, which is critical for long-term dental health [10]. Moreover, the dose-dependent effect observed with varying concentrations of white tea extract indicates that optimizing the extract's concentration could be essential for maximizing its benefits in dental materials. Higher concentrations of white tea extract, such as the 10% used in this study, appear to provide more robust protection against collagen degradation and greater improvement in mechanical properties. This aligns with other research findings, which suggest that the concentration of bioactive compounds in natural extracts directly influences their efficacy in enhancing the properties of dental materials [11]. Future studies should explore the optimal concentration range to balance efficacy with material stability and handling properties [12].The implications of these findings extend beyond the immediate application of white tea extract in calcium silicate cement. They suggest a broader potential for incorporating natural antioxidants into various dental materials to enhance their performance and longevity. This approach could lead to the development of more durable, biocompatible and effective dental restoratives, particularly in areas where the preservation of dentin and prevention of collagen degradation are critical. The integration of such natural extracts could pave the way for more sustainable and health-promoting dental treatments, in line with the growing emphasis on biomimetic and regenerative dentistry [13]. Further research is needed to investigate the long-term effects of such materials in clinical settings, as well as their potential to interact synergistically with other dental treatments [14-16].

## Conclusion:

Data shows that by strengthening dentin's flexural strength and slowing the deterioration of collagen, adding white tea extract to calcium silicate cement may increase the lifetime and durability of dental restorations. To investigate the long-term effects of white tea extract on dentin in clinical settings, further research is necessary.

## Figures and Tables

**Table 1:** Flexural strength of dentin specimens (MPa)

**Group**	**Mean Flexural Strength (MPa)**	**StandardDeviation (MPa)**
Group A (Control)	100.5	5.3
Group B (Conventional CSC)	105.8	4.7
Group C (5% White Tea Extract)	115.2	6.1
Group D (10% White Tea Extract)	120.4	5.9

**Table 2:** Hydroxyproline release (μg/mL) Over 30 Days

**Group**	**Day 1**	**Day 7**	**Day 14**	**Day 30**
Group A (Control)	8.2	15.4	18.7	20.1
Group B (Conventional CSC)	7.6	14.2	17.3	18.9
Group C (5% White Tea Extract)	5.1	9.8	11.7	12.4
Group D (10% White Tea Extract)	4.7	8.6	10.2	10.8
